# Two-dimensional mathematical framework for evaporation dynamics of respiratory droplets

**DOI:** 10.1063/5.0064635

**Published:** 2021-10-01

**Authors:** Sreeparna Majee, Abhishek Saha, Swetaprovo Chaudhuri, Dipshikha Chakravortty, Saptarshi Basu

**Affiliations:** 1 Department of Mechanical Engineering, Indian Institute of Science, Bengaluru, Karnataka 560012, India; 2 Department of Mechanical and Aerospace Engineering, University of California San Diego, La Jolla, California 92093, USA; 3 Institute for Aerospace Studies, University of Toronto, Toronto, Ontario M3H 5T6, Canada; 4 Department of Microbiology and Cell Biology, Indian Institute of Science, Bengaluru, Karnataka 560012, India

## Abstract

In majority of pandemics in human history, respiratory bio-aerosol is the most common route of transmission of diseases. These tiny droplets ejected through mouth and nose from an infected person during exhalation process like coughing, sneezing, speaking, and breathing consist of pathogens and a complex mixture of volatile and nonvolatile substances. A cloud of droplets ejected in such an event gets transmitted in the air, causing a series of coupled thermo-physical processes. Contemplating an individual airborne droplet in the cloud, boundary layers and wakes develop due to relative motion between the droplet and the ambient air. The complex phenomenon of the droplet's dynamics, such as shear-driven internal circulation of the liquid phase and Stefan flow due to vaporization or condensation, comes into effect. In this study, we present a mathematical description of the coupled subprocesses, including droplet aerodynamics, heat, and mass transfer, which were identified and subsequently solved. The presented two-dimensional model gives a complete analysis encompassing the gas phase coupled with the liquid phase responsible for the airborne droplet kinetics in the ambient environment. The transient inhomogeneity of temperature and concentration distribution in the liquid phase caused due to the convective and diffusive transports are captured in the 2D model. The evaporation time and distance traveled by droplets prior to nuclei or aerosol formation are computed for major geographical locations around the globe for nominal-windy conditions. The model presented can be used for determining the evaporation timescale of any viral or bacterial laden respiratory droplets across any geographical location.

## I. INTRODUCTION

In the context of awareness and understanding the spread of the deadly SARS-CoV-2 virus, which is responsible for the COVID-19 pandemic, many research studies^1–4^ have shown that airborne droplet is the main route of disease transmission. Other viruses and bacteria-like influenza virus,^5^ *Mycobacterium tuberculosis,*^6^ and *Pseudomonas aeruginosa*^7^ also transmit through respiratory aerosol causing the spread of disease. Studies have also proven that aerosols are involved in the spread of SARS, MERS, H1N1, and many other diseases.^8–10^ Wells^11,12^ was the pioneer to provide one of the first mechanistic description of the disease transmission route through respiratory droplets. The pathogens are transmitted from infected to healthy person through droplet cloud, produced by the respiratory actions during cough, sneeze, speech, etc. This cloud consists of droplets of varying size^13^ where larger droplets settle on ground due to gravity,^4^ while smaller droplets or aerosols can travel larger distances causing elongated exposure. Recent works on COVID-19 have shown that respiratory events such as cough and sneeze are predominantly made of aerosol particles contained inside a multiphase turbulent gas (a puff) cloud that entrains ambient air.^4,14,15^

Aerosols are unstable accumulation of liquid or solid particles of different sizes that constantly varies with external velocity field, gravitational settling, temperature, and mass transfer with the surrounding gas medium. The particles in a bio-aerosol are generally 0.3–100 μm in diameter, and its ability to remain in the air and travel larger distances is of primary concern.^16^ Moreover, Gorbunov^17^ suggested that aerosols can ensemble in regularly used public spaces mainly hospitals, public transports, and supermarkets, facilitating direct human-to-human respiratory bio-aerosol transmission. Enhanced transmission rate in confined spaces is also discussed by several researchers.^18–20^ To minimize the transmission, the world scientific and medical community suggests the use of face masks and social distancing of 2 m.^21–23^ However, this social distancing norm of 2 m does not hold for aerosol transmission,^17^ depending on various atmospheric conditions.

One of the crucial factors in the transport phenomena of these respiratory droplets in the air is the evaporation of the respiratory droplets, which is dependent on the ambient temperature and relative humidity. As presented in review by Pica and Bouvier,^24^ pathogen infection spread and transmission can highly depend on factors such as relative humidity, ambient temperature, and surrounding airflow. However, the composition of respiratory fluid is highly complex, containing organic compounds, salts, proteins, surfactants, and pathogens (virus or bacteria).^25^ Therefore, the evaporation dynamics of these complex fluid droplets in the air are complicated. These complex fluid droplets behave significantly different from pure water droplets^4^ as the nonvolatile components present in the fluid affect evaporation dynamics.^26^ As shown by Vejerano *et al.,*^26^ respiratory droplet primarily contains ∼1% (w/w) NaCl dissolved in water (solvent). Moreover, it is also noted that the pathogens (virion) do not affect the evaporation or precipitation dynamics as shown in experimental data^14^ and thus neglected in modeling the global transport processes.

Furthermore, during any respiratory event, the droplets are exhaled in the ambient, consisting of a large volume of air, which initially act as a turbulent jet but gradually transforms into a buoyant puff.^27^ As the droplets within the cloud transmit through jet and puff, the current model incorporates the evolution of these respiratory jet and puff in the mathematical description for realistic approach.

Because of the complexities described above, in this paper, we have numerically simulated a two-dimensional model based on an isolated droplet from the cloud, assuming one-way coupling between the droplet and surrounding air. In this case, the droplet–droplet interaction or effects of surrounding droplets are neglected as the liquid phase volume (droplets) is too small to affect the gas phase (air) for respiratory events. More popular approach with the homogeneous 0D (zero) model^4^ assumes that the droplet is homogenous, and as such, no liquid phase transport is considered. As an important modification to the homogeneous model, we have solved a detailed transport model for liquid phase inside the droplet and investigated a spatiotemporal solute–solvent level heat and mass transfer, which affects the evaporation rate and further nucleation of the droplet. The current model provides a reasonably agreeable results validated with experimental and the homogeneous model data.^4^ Similar to the homogeneous model, here, the temporal or spatial evolution of flow in gas phase is not solved in detail, as it requires a large scale either direct numerical transport (DNS) or large-eddy simulations (LES) to capture the turbulent characteristics.^28–33^ Although such perspective could provide a greater insight, they need more computational resources and simulations for varying ambient conditions, which are difficult to achieve. These DNS and LES studies, on the other hand, do not solve the liquid phase transport due to prohibitive computational costs. Thus, findings of our study with detailed liquid phase transport complement the previous approaches. We will show that varying atmospheric temperature and relative humidity based on the geographical location, the two-dimensional model successfully predicts the corresponding evaporation time until nucleation and distance traveled by the aerosol in nominal-windy conditions globally. However, the axial distance increases by multiple folds having a constant evaporation time when average wind velocity is considered.

## II. TWO-DIMENSIONAL MODELING OF RESPIRATORY DROPLET

**FIG. 1.**
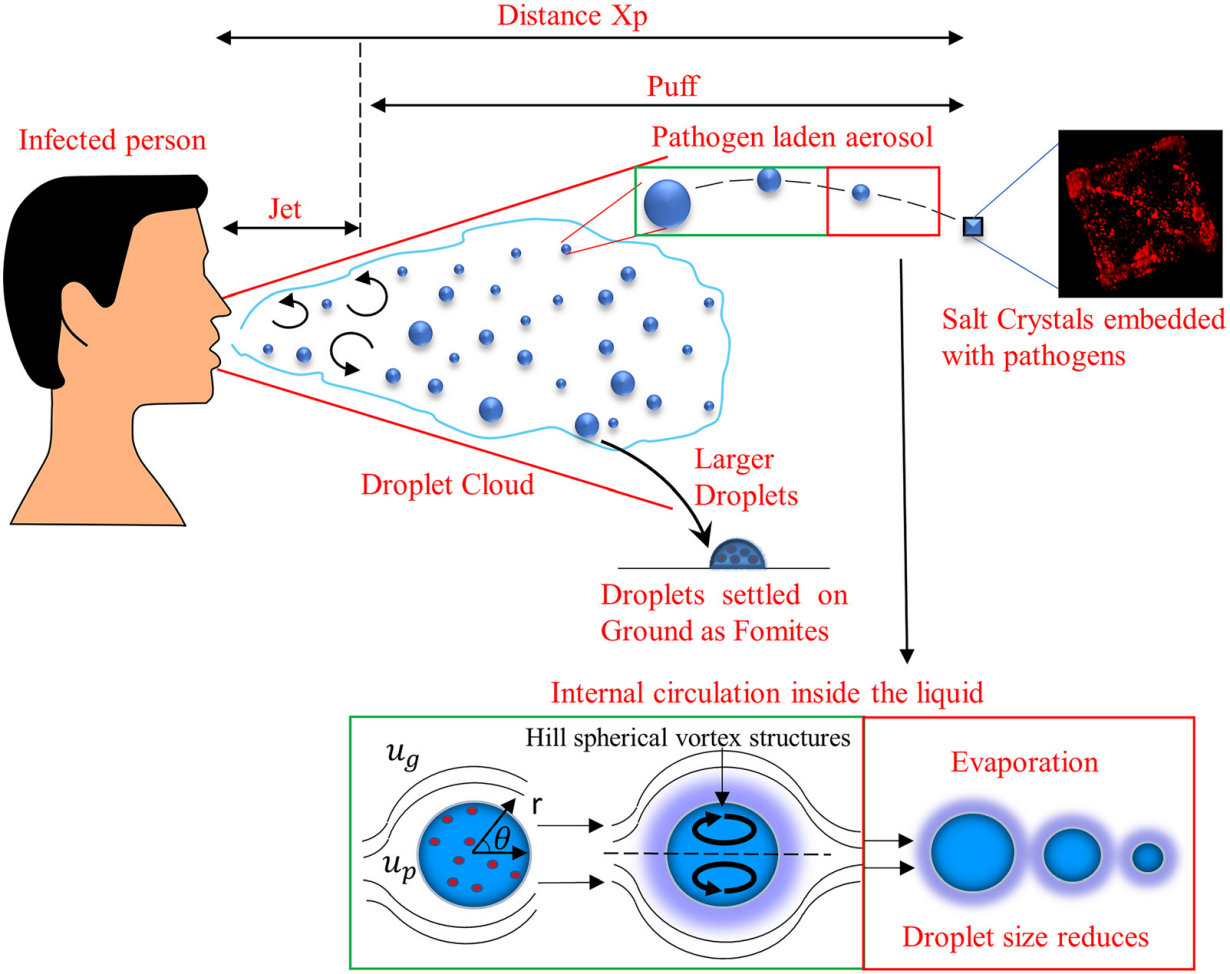
A Schematic of evaporation dynamics of pathogen laden respiratory droplets. (The crystal image is reproduced with the permission from Basu *et al.*, “Insights on drying and precipitation dynamics of respiratory droplets from the perspective of COVID-19,” Phys. Fluids **32**, 123317 (2020). Copyright 2020 AIP publishing.

As mentioned in the introduction, during any respiratory episode such as breathing, sneezing, or coughing, bio-aerosols are ejected in clouds which initially for a short duration evolve as turbulent jet and then transform to a puff as discussed in.^4^ These respiratory droplets can either remain in the air or settle on a surface as fomites. Evaporation of these bio-aerosols dispersed with the cough-cloud is complicated due to the heat and mass transfer of the solvent and nonvolatile salts (predominantly NaCl) present in our saliva (Fig. 1). These conditions strongly influence the evaporation dynamics of a droplet. The two-dimensional model gives an absolute analysis encompassing the gas phase coupled with the liquid phase responsible for complex evaporation phenomenon of the airborne droplet kinetics in the ambient environment. The modeling framework as advocated by Abramzon and Sirignano^34^ has been adopted here. They introduced a new approximate model of vaporization of a moving fuel droplet extending the classical droplet vaporization model including important effects as variable physical properties and non-unitary Lewis number in the gas phase, influence of the Stefan flow (blowing) on heat and mass transfer, and the effect of the transient liquid heating inside the internally circulating droplet. The schematic of the lifetime of an isolated exhaled droplet in the ambient atmosphere is given in Fig. 1.

The model involves three-part analysis considering the motion of the droplet in the ambient, gas phase around the droplet and liquid phase of the droplet. The motion of the droplet is solved to determine the velocity of the droplet and its trajectory. The droplet trail in the ambient air is a function of ambient temperatures and relative humidity that the droplets experience. Here, we assumed the cough jet velocities to be dominant in the axial direction; therefore, the radial velocity component of the cough jet is neglected.

The mean axial location and velocity together with mean radial spread of the droplet gas phase divided into transient turbulent jet and puff can be expressed,^4,15,27,35,36^ respectively, as

$$\begin{array}{c} x_{j}\left(t\right)={\left(\frac{12}{K}\right)}^{\frac{1}{2}}{\left(U_{j,0}R_{j,0}\right)}^{1/2}t^{1/2},\\ U_{j}\left(t\right)=\frac{6U_{j,0}R_{j,0}}{Kx_{j}\left(t\right)},\\ R_{j}\left(t\right)=R_{j,0}+\frac{x_{j}\left(t\right)-x_{j,0}}{5} \end{array}$$ (1)

and

$$\begin{array}{c} x_{pf}\left(t\right)=\frac{3m}{a}R_{pf}\left(t\right),\\ U_{pf}\left(t\right)=U_{pf,0}{\left(\frac{3mR_{pf,0}}{4aU_{pf,0}t}\right)}^{3/4},\\ R_{pf}\left(t\right)=R_{pf,0}{\left(\frac{4aU_{pf,0}t}{3mR_{pf,0}}\right)}^{1/4} \end{array}$$ (2)

where subscripts j and pf mean jet and puff, respectively. R_0_ and U_0_ are the initial radius and axial velocities at a distance x_0_. For a fleeting turbulent jet, K is a characteristic constant based on,^37^ which is estimated to be 0.457. Since jet cloud initiates from the mouth itself so at t = 0, the radius of the jet event is approximated to be similar to human mouth radius, 14 mm. However, the ejection velocity of the jet cloud U_j,0_ depends on the type of respiratory event. Taking examples of various respiratory event, U_j,0_ for sneezing is on the order of 40 m/s; for coughing, it is approximately 10 m/s, while for speaking and singing, it is about 2–3 m/s. Thereafter, to scale the turbulent puff, the characteristic constants considered are a ≈ 2.25 and m = (x_p,0_a)/(3R_p,0_) ≈ 4.^35^

However, the current paper is restricted to only cough events (U_j,0_ = 10 m/s), but can easily be adapted for other respiratory events. A significant factor for the jet event must be noted that for a single cough event, the ejection of jet lasts only for approximately 1 s.^36^ Beyond this time, the turbulent jet undergoes transition to the turbulent puff together constituting the gas phase described as

$$\begin{array}{c} U_{g}=\{\begin{array}{l} U_{j}\left(t\right)\;\;\;\;\;\;\;\;\;\;\;\;t\leq 1\;s\\ U_{pf}\left(t\right)\;\;\;\;\;\;\;\;\;t>1\;s \end{array}\\ R_{g}=\left\{\begin{array}{l} R_{j}\left(t\right)\;\;\;\;\;\;\;\;\;\;\;\;t\leq 1\;s\\ R_{pf}\left(t\right)\;\;\;\;\;\;\;\;\;\;t>1\;s. \end{array}\right. \end{array}$$ (3)

### A. Gas phase

The momentum equations responsible for the droplet motion and the droplet evaporation rate are defined as

dXpdt=UpdUpdt=3CD8rsρgρLUg−UpUg−UpdZpdt=VpdVpdt=−3CD8rsρvρLVp2+ρL−ρvρLg}, (4)

$$\frac{dr_{s}}{dt}=-\frac{\dot{m}}{4\pi \rho_{L}r_{s}^{2}},$$ (5)

where $X_{p}$ is the horizontal displacement of the exhaled droplet and $U_{p}$ is its instantaneous velocity. $V_{p}$ and $Z_{p}$ are the droplet velocity and displacement in the traverse direction, respectively. Here, $U_{g}$ and $\rho_{g}$ are the droplet gas phase velocity and density of the gas phase; $r_{s}$ is instantaneous radius of the droplet; $\rho_{L}$ is liquid phase densities, $\mu_{g}$ is gas phase dynamic viscosity, and $C_{D}$ is the drag co-efficient, which can be taken as 24/ ${Re}_{p}$ for the gas phase Reynolds number, ${Re}_{p}=2\rho_{v}\left|U_{g}-U_{p}\right|r_{s}/\mu_{g}<30,$ which is mostly less than 0.1 for respiratory droplets. The model assumes that the respiratory jet and the droplets within that are exhaled in equivalent velocity. Therefore, to refrain from the singularity in calculating CD, the initial droplet velocity is considered as 99% of the jet velocity. The variation in humidity and temperature within the droplet cloud is not regarded in the current approach, while it can be found in Ref. 15.

The average temperature is chosen as *T*_mean_ = (2 $T_{s}$ + $T_{\infty }$)/3 as suggested by Hubbard *et al.,*^38^ where $T_{s}$ and $T_{\infty }$ are the droplet surface temperature and temperature of the gas phase at far-field, respectively. The solution of Eqs. (4) and (5) is determined by the vaporization rate. This vaporization rate is obtained from the coupled liquid and vapor phase analyses, where the solution for vapor phase surrounding the liquid droplet is assumed to be in a quasi-steady-state condition. Thus, the expressions for vaporization mass flux are given as (heat and mass transfer limits)

$$\dot{m}=2\pi {\rho_{v}D}_{\upsilon }r_{s}{Sh}^{*}\log\left(1+B_{M}\right),$$ (6)

$$\dot{m}=2\pi {\rho_{v}\alpha }_{g}r_{s}{Nu}^{*}\log\left(1+B_{T}\right),$$ (7)

where $\dot{m}$ is the rate of change of droplet water mass due to evaporation, $\rho_{v}$ is density of water vapor, and $D_{\upsilon }$ is the binary diffusivity of water vapor in the air, and $\alpha_{g}$ is the thermal diffusivity of surrounding air. Here, ${Nu}^{*}$ and ${Sh}^{*}$ are the corrected Nusselt and Sherwood numbers, respectively, according to the relative changes of film thickness due to surface blowing effect. The Spalding heat and mass transfer numbers are defined as $B_{M}$ = $(Y_{W,s}-Y_{W,\infty })\;/\left(1-Y_{W,s}\right)$ and $B_{T}$ = $Cp,l(T_{\infty }-T_{s})/{(h}_{fg}+Q_{l}/\dot{m})$. Here, $Y_{W}$ is mass fraction of water vapor, while subscript s and ∞ denote location at droplet surface and at far field, respectively. The subscripts W, A, and N will denote water, air, and salt, respectively. Cp,l, and $h_{fg}$ are the specific heat and specific latent heat of vaporization of the droplet liquid, and $Q_{l}$ is the heat transferred into the liquid. In the case for respiratory droplets, the evaporation kinetics gets affected as the nonvolatile solutes (1% w/w NaCl) suppress the vapor pressure at the droplet surface. This is taken into account by considering an ideal solution obeying the Raoult's Law,^39^ $P_{\mathrm{vap}}\left(T_{s},\;\chi_{W,s}\right)=\chi_{W,s}P\mathrm{sat}(T_{s})$, where $\chi_{W,s}$ is the mole fraction of evaporating solvent (here water) at droplet surface in the liquid phase^40^ and $\chi_{W,s}=1-\chi_{N,s}$. Following the above considerations, the vapor concentrations at droplet surface and at far field can be expressed as

$${\;Y}_{W,s}=\frac{P_{\mathrm{vap}}(T_{s,\;\chi W,s})M_{W}}{P_{\mathrm{vap}}\left(T_{s,\chi W,s}\right)M_{W}+{(1-P_{\mathrm{vap}}(T_{s,\chi W,s}))M}_{A}},$$ (8)

$${\;Y}_{W,\infty }=\frac{{(RH)P}_{\mathrm{sat}}(T_{\infty })M_{W}}{{(RH)P}_{\mathrm{sat}}(T_{\infty })M_{W}+{(1-{(RH)P}_{\mathrm{sat}}(T_{\infty }))M}_{A}}.$$ (9)

After determining the mass vaporization rate and the heat transferred into the droplet interior $Q_{l}$, the temperature and concentration distributions inside the vaporizing droplet are solved with the help of governing equations for conservation of mass and energy.

### B. Liquid phase

Now, if we look inside the liquid phase of the drop, moving droplets produces convective motion due to the intensive liquid circulation caused by the surface friction. Two-dimensional flow is assumed by considering internal flow structures described by the well-known Hill's spherical vortex.^41^

The flow around the droplet is assumed to be axisymmetric such as the flow characteristics are independent of θ but varies with time. Using the Hill spherical vortex solution, the radial and angular velocity field in the spherical coordinate system $\left(r,\theta \right)$ inside the moving evaporating droplet is given as

$${\;V}_{r}=-U_{s}\left(1-\frac{r^{2}}{{r_{s}}^{2}}\right)\text{cos }\theta ,$$ (10)

$${\;V}_{\theta }=U_{s}\left(1-2\frac{r^{2}}{{r_{s}}^{2}}\right)\text{sin }\theta ,$$ (11)

where $U_{s}=(1/32)\left(U_{g}-U_{p}\right)(\mu_{g}/\mu_{l}){Re}_{p}C_{F}$ is the velocity of the liquid at the liquid–vapor interface and is calculated by the continuity of the shear stress across the interface; $\mu_{l}$ is liquid phase dynamic viscosity. Using the correlation given by Renksizbulut and Yuen^42^ that the Stefan flow reduces the friction drag coefficient by a factor ( $1+B_{M}$), the skin friction coefficient for an evaporating sphere is given by

$${\;C}_{F}=\frac{12.69{{Re}_{p}}^{-2/3}}{1+B_{M}}.$$ (12)

The thermal energy equation inside the circulating and evaporating droplet is obtained by non-dimensionalizing the transient convection–diffusion equation in polar coordinates:^43^

$$\;\;\;\;\bar{r_{s}^{2}}\frac{\partial \bar{T}}{\partial \tau }+\left(0.5\;{Pe}_{L}\bar{V_{r}}\bar{r_{s}}-\beta \eta \right)\frac{\partial \bar{T}}{\partial \eta }+0.5{Pe}_{L}\frac{\bar{V_{\theta }}\bar{r_{s}}}{\eta }\frac{\partial \bar{T}}{\partial \theta }=\frac{1}{\eta^{2}}\frac{\partial }{\partial \eta }\left(\eta^{2}\frac{\partial \bar{T}}{\partial \eta }\right)+\frac{1}{\eta^{2}\;\text{sin}\;\theta }\frac{\partial }{\partial \theta }\left(\;\text{sin}\;\theta \frac{\partial \bar{T}}{\partial \theta }\right).$$ (13)

Here, $\bar{r_{s}}=r_{s}/r_{0}$ is the dimensionless radius of the droplet; $\eta =r/r_{s}$ is the dimensionless radial coordinate; $\bar{V_{r}}=V_{r}/U_{s}$ and $\bar{V_{\theta }}=V_{\theta }/U_{s}$ are the dimensionless radial and angular velocities, respectively; $\bar{T}={(T-T}_{0)}/T_{0}$ is the dimensionless temperature; $\tau =\alpha_{l}t/r_{0}^{2}$ is the dimensionless time; $\beta =0.5\partial \bar{r_{s}}/\partial \tau$ is the dimensionless parameter proportional to the surface regression rate of the droplet; and $\alpha_{l}$ is thermal diffusivity of the liquid phase. The initial and boundary conditions are given as

$$\begin{array}{c} \tau =0\to \;\bar{T}=0\\ \eta =1,\;\{\begin{array}{l} \frac{\partial \bar{T}}{\partial \theta }=0\\ \int_{0}^{\pi }\frac{\partial \bar{T}}{\partial \eta }\text{sin}\;\theta \;d\theta =\frac{Q_{L}}{2\pi r_{s}k_{l}T_{0}} \end{array}\\ \theta =0,\;\pi \to \frac{\partial \bar{T}}{\partial \theta }=0. \end{array}$$ (14)

Similarly, the conservation of species equation can be written as^43^

$$\begin{array}{c} {Le}_{L}\bar{r_{s}^{2}}\frac{\partial {\bar{Y}}_{N}}{\partial \tau }+\left(0.5\;{Pe}_{L}{Le}_{L}\bar{V_{r}}\bar{r_{s}}-{Le}_{L}\beta \eta \right)\frac{\partial {\bar{Y}}_{N}}{\partial \eta }+0.5{Pe}_{L}{Le}_{L}\frac{\bar{V_{\theta }}\bar{r_{s}}}{\eta }\frac{\partial {\bar{Y}}_{N}}{\partial \theta }\\ =\frac{1}{\eta^{2}}\frac{\partial }{\partial \eta }\left(\eta^{2}\frac{\partial {\bar{Y}}_{N}}{\partial \eta }\right)+\frac{1}{\eta^{2}\;\text{sin}\;\theta }\frac{\partial }{\partial \theta }\left(\;\text{sin}\;\theta \frac{\partial {\bar{Y}}_{N}}{\partial \theta }\right) \end{array}.$$ (15)

Here, ${\bar{Y}}_{N}=(Y_{N}-Y_{N,0})/Y_{N,0}$ is the normalized mass fraction of the solute. The corresponding initial and boundary conditions are

$$\begin{array}{c} \tau =0\to {\bar{Y}}_{N}=0\\ \begin{array}{c} \eta =1,\;\{\begin{array}{l} \frac{\partial {\bar{Y}}_{N}}{\partial \theta }=0\\ \int_{0}^{\pi }\frac{\partial {\bar{Y}}_{N}}{\partial \eta }\text{sin}\;\theta \;d\theta =\frac{\dot{m}}{2\pi \rho_{L}r_{s}D_{v,za}Y_{N,0}} \end{array}\;\\ \theta =0,\;\pi \to \frac{\partial {\bar{Y}}_{N}}{\partial \theta }=0, \end{array} \end{array}$$ (16)

$K_{l}$ is the thermal conductivity of the liquid phase and $D_{v,za}$ is the mass diffusivity of NaCl in water. ${Pe}_{L}$ and ${Le}_{L}$ are the Peclet number calculated based on liquid properties and Lewis number of the liquid phase, respectively. It is to be noted we assumed that temperature and concentration along with droplet surface are uniform; thus, the Marangoni stress terms are not considered in our equation. At the onset of the respiratory event (t = 0), the gas phase velocity of the bio-aerosol cloud is assumed to be 10 m/s and a radius of the cloud as 14 mm, which is the mean human mouth opening.

The numerical simulation has been carried out by solving Eqs. (4)–(16), both for external vapor and internal liquid regions. However, the internal liquid phase solution requires the boundary conditions (14) and (16), obtained by solving the vapor phase. Accordingly, Eq. (4) is solved by numerical integration method using forward marching scheme. The mass flux $\dot{m}$ calculated from Eq. (6) generates the solution of Eq. (5); hence, the instantaneous droplet radius can be computed by a forward marching scheme. This mass flux is subsequently used as the boundary condition for the conservation of species equation. Following the completion of the mass transfer part of the vapor phase, heat transfer in the vapor phase is formulated implicitly.^43^ The calculated surface heat flux $Q_{L}$ is employed in the boundary condition for the energy equation of the liquid phase, similar to the mass transfer solution. A detailed stepwise algorithm of the numerical procedure ^34^ is given in the supplementary material with diagram representing the dependence of each equation as Fig. S1.

After obtaining the boundary conditions from the vapor phase, the thermal energy Eq. (13) and species Eq. (15) along with the boundary conditions (14) and (16) in the liquid phase are computed numerically by fully implicit iterative finite difference scheme called standard second-order Peaceman–Rachford ADI method,^44^ which is a double time step algorithm. The resulting spatiotemporal approximations considered in this method are of second order, and the finite-difference grid spacings ( Δη, Δθ) are uniform. Due to the consideration of an implicit scheme, this method is unconditionally stable, where Δτ = 0.0057, Δη = 0.05, and Δθ = 0.157 were taken for the entire simulation process. Moreover, it is investigated that, if we further decrease Δτ, no significant change in the numerical results is exhibited.

## III. RESULTS AND DISCUSSION

**FIG. 2.**
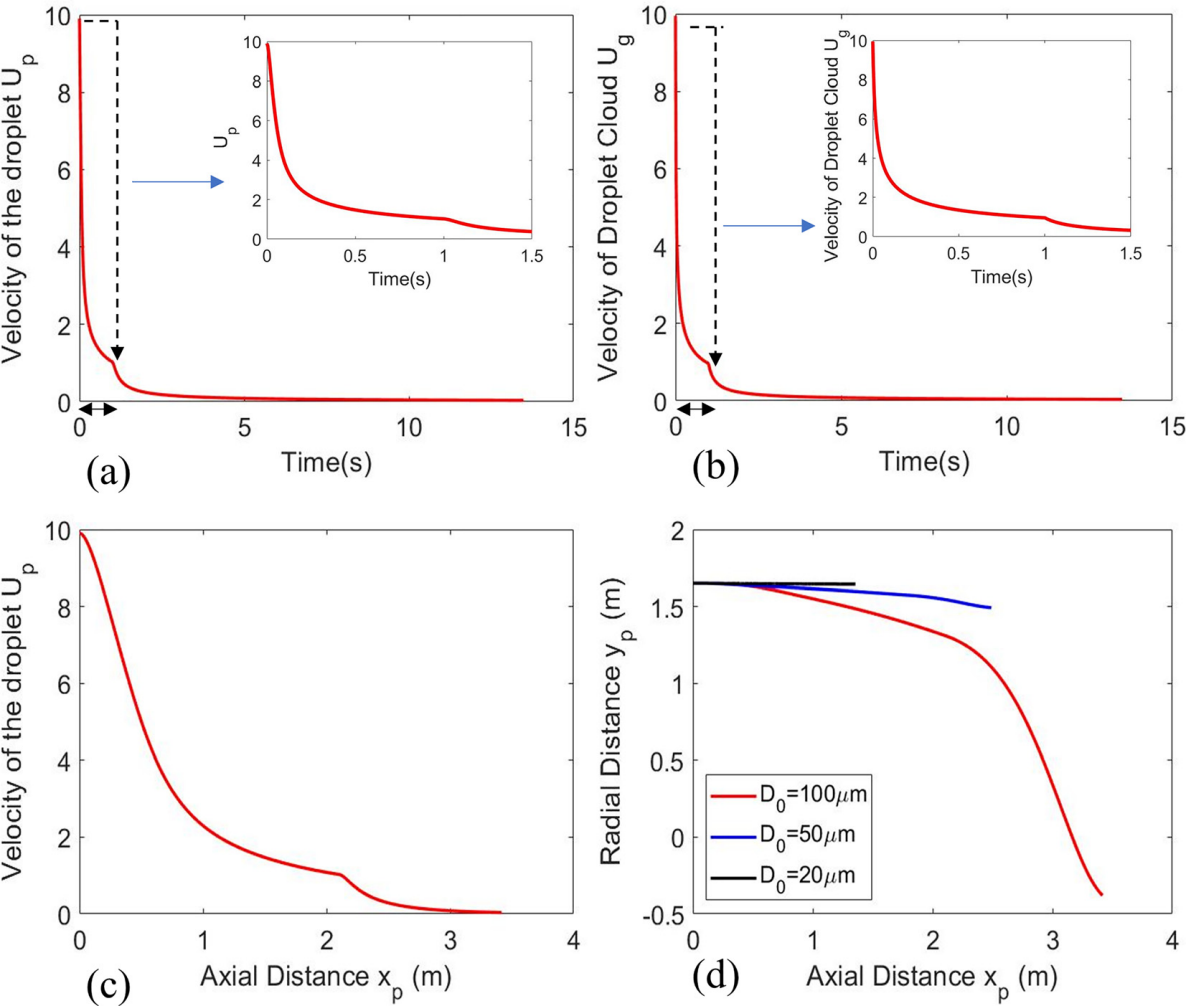
(a) Velocity of droplet ( $D_{0}=100\;\mu m$) with respect to time including an inset figure from time 0–1.5 s and (b) velocity of the droplet cloud (jet and puff) with respect to time including an inset figure from time 0 to 1.5 s, (c) velocity of droplet along with axial distance traveled by the droplet during the period of evaporation, and (d) trajectory of the droplet. Initial T_∞_ and RH_∞_ is 28 °C and 48%, respectively.

Due to the jet and consecutive puff profile of the droplet cloud, it affects both droplet velocity and vapor phase velocity. As discussed earlier, the injection of droplet velocity is very close to the injection of respiratory jet, the droplet velocity U_p_ = 0.99U_g_, and subsequently, the difference becomes smaller due to drag. This provides a similar profile of the droplet velocity and vapor phase velocity with time given in Figs. 2(a) and 2(b). The kink in both the figures at t = 1 s arises from the transition of gas phase solution from the jet to the puff behavior as shown in Eq. (3). Both the velocities in the puff phase slowly approach to zero until the final evaporation time before nucleation of the droplets is reached at 28 °C ambient temperature and 48% relative humidity. Figure 2(c) depicts the droplet velocity ( $D_{0}$ = 100 μm) along with the axial direction $x_{p}$ of the droplet moving in the air. This gives us the idea that a droplet with initial diameter 100 μm can travel upto 3.5 m in non-windy scenario at the above-mentioned ambient temperature and humidity conditions. The trajectory of a single droplet with three different initial diameters moving in the air is generated in Fig. 2(d). The starting point of the droplet is made at a radial location equivalent to the height of the mouth in an average human at 1.65 m. The bigger droplet ( $D_{0}$ = 100 μm) axially travel upto certain distance due to the jet flow and gradually fall in the ground due to gravity. However, smaller droplets ( $D_{0}$ = 50 and 20 μm) evaporate in the air and never settles on the ground. These droplets after nucleation may travel longer distances enhancing the spread of disease. Furthermore, under windy conditions, such as in locations of average wind speed of 2 m/s, the axial spread of the droplets ( $D_{0}$ = 100 μm) increases 20 times than in a non-windy condition, maintaining identical evaporation time. This refutes the standard social distancing norm of 2 m or 6 feet as bio-aerosol can spread up to several meters depending on the size of aerosol and wind speed of the location. Moreover, in this study, the Weber number ≪ 1, considering the surface tension of water (very low salt concentration). Thus, breakup or deformation of the droplet is neglected. Apart from the locations close to the mouth, the number density of the droplets in exhaled volume is rather small. Hence, the entire system can be considered to be dilute. As the surrounding jet-puff of the air expands in diameter and the droplets disperse, the probability of collision and subsequent coalescence should be rare.

**FIG. 3.**
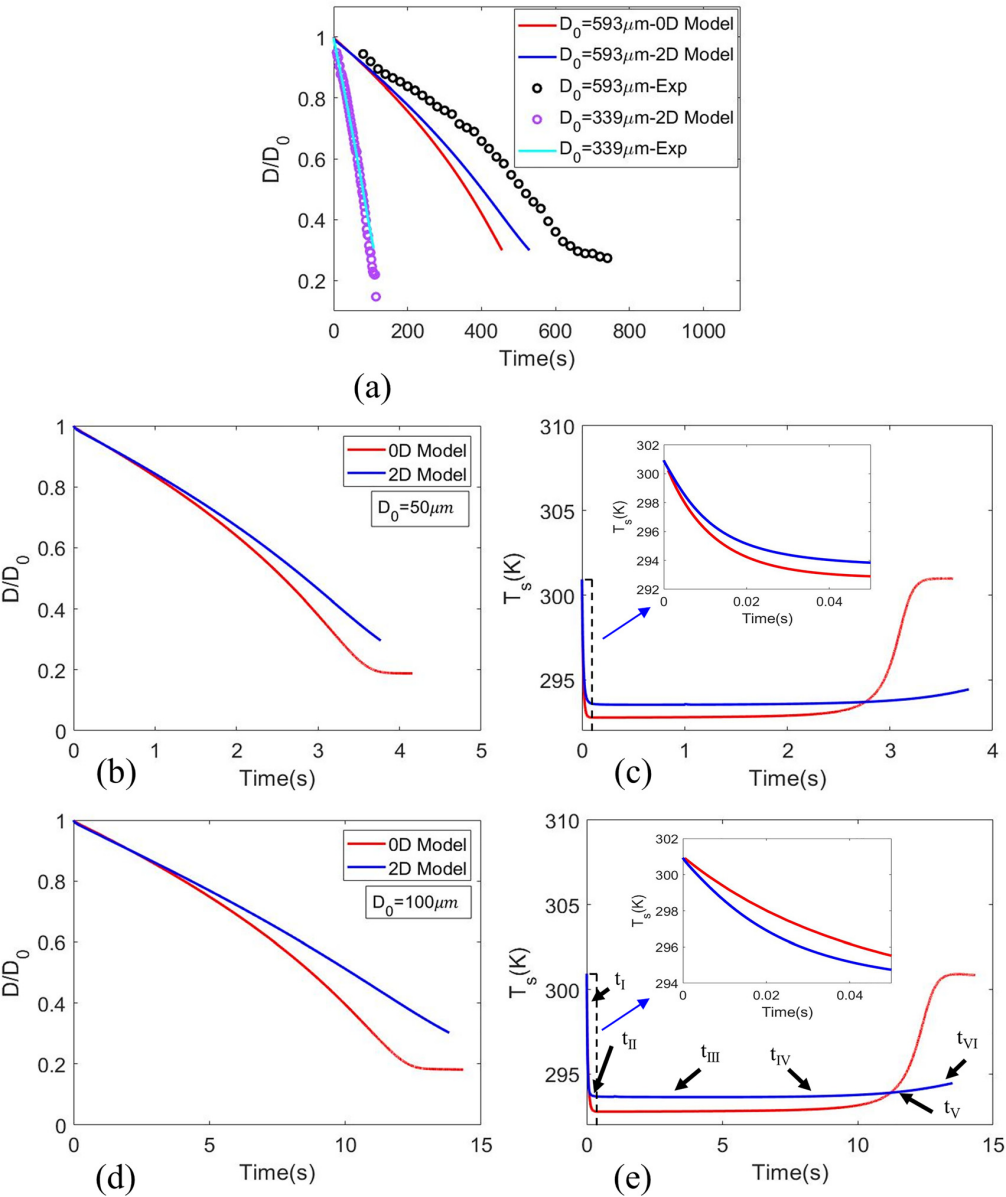
(a) Comparison of experiments and simulations of normalized droplet diameter as a function of time for salt–water solution droplet with 48%RH, $D_{0}=593\;\mu m$ and 35%RH, $D_{0}=339\;\mu m$. Comparison of 0D and 2D modeling of normalized (b) and (d) droplet diameter and (c) and (e) droplet surface temperature as a function of time surrogate respiratory droplets solution droplet including an inset figure from time 0 to 0.05 s. Initial droplet diameter: (b) and (c) 50 mm and (d) and (e) 100 mm. The ambient was T_∞_ = 28 °C, RH_∞_ = 48%, and initial droplet temperature was T_s,0_ = 30 °C. The stages of tI to tVI are correlated with the stages of Fig. 5.

For validation of the numerical model, few oriented experiments were conducted using an isolated levitated droplet evaporation of 1% w/w NaCl solutions to replicate the actual respiratory droplets in 28 ± 0.2 °C ambient temperature and 48 ± 0.002% relative humidity.^4^ Figure 3(a) exhibits a comparison between experimental outcome, the 0D model,^4^ and the current 2D model for initial droplet diameter as 593 μm. The model shows reasonably good agreement with experimental results and 0D model with inconsequential deviations. The current model is terminated immediately with the trigger of precipitation as solute concentration at the surface reaches the critical value $Y_{N,S}=0.393.$^45^ Moreover, the validation of the results is also done using varying grid sizes as shown in supplementary material Fig. S2.

In Figs. 3(b) and 3(e), comparisons between the 0D and 2D model are presented for two initial diameters ( $D_{0}$ = 50 and 100 μm). The ambient T_∞_ = 28 °C and RH_∞_ = 0.48 were maintained, and initial droplet temperature was T_s,0_ = 30 °C. Due to the minimal contrast in the initial phase of injection velocity of the droplets and ambient gas-phase velocity (U_p,0_ = 0.99U_j,0_), followed by similar profile [Figs. 2(a) and 2(b)], induces a momentary Hills vortex and hence, we expect less inhomogeneity inside the droplet. Thus, the $D/D_{0}$ plot with time for both initial diameters in the 2D model agrees with the 0D homogeneous model. The 2D model predicts a slightly higher wet bulb temperature compared to 0D model. Furthermore, transport of heat and mass from the surface of the droplet to the center indeed takes finite time (an aspect which is ignored in homogeneous model). Nevertheless, the comparison illustrates the fact that the 0D model^4^ demonstrates reasonably good predictive capability. However, under a different circumstance (with large thermal and velocity difference) such as high-speed ambience or droplet injection in plasma jet, the relative velocity is enhanced. Such high relative velocity and temperature differences will lead to significant errors in the 0D homogeneous model in which the current 2D model can handle efficiently.

As expected, for T_s,0 _> T_∞_, the droplet undergoes rapid cooling and eventually reach a steady state limit (wet bulb) as the evaporation progresses. As shown in Figs. 3(d) and 3(e) in both droplet sizes, both models predict a wet bulb temperature around 21 °C (2D model predicts slightly higher temperature), a limit which ensures T_s,0_ < T_∞_. Consequently, after attaining the wet-bulb limit, we have positive heat transfer from the ambient to the droplet surface, which is used completely for evaporating the droplet. For a respiratory droplet with the dissolved nonvolatile salts, the evaporation is dependent on the solvent (water here) mole fraction at the droplet surface. Due to evaporative mass loss during evaporation, the solvent concentration is reduced at the droplet surface, while the mole fraction of the solute increases. Once the mole fraction of the solute reaches the critical supersaturation limit, precipitation begins.

Tables I and II, in Ref. 46, represent the ambient temperature and relative humidity of different cities for winter and summer/monsoon, respectively. A–F represents outdoor conditions of regions like Berlin, Delhi, Kolkata, London, Nairobi, and New York, respectively.

**TABLE I.** Ambient temperature and relative humidity for different cities in winter.

Cities	Ambient temperature (°C)	Relative humidity (%)
A	0.5	84
B	13	67
C	19	64
D	5	86
E	19.7	59
F	−1	66

**TABLE II.** Ambient temperature and relative humidity for different cities in summer/monsoon.

Cities	Ambient temperature (°C)	Relative humidity (%)
A	21	67
B	33	52
C	30	80
D	21	63
E	18	61
F	24	38

**FIG. 4.**
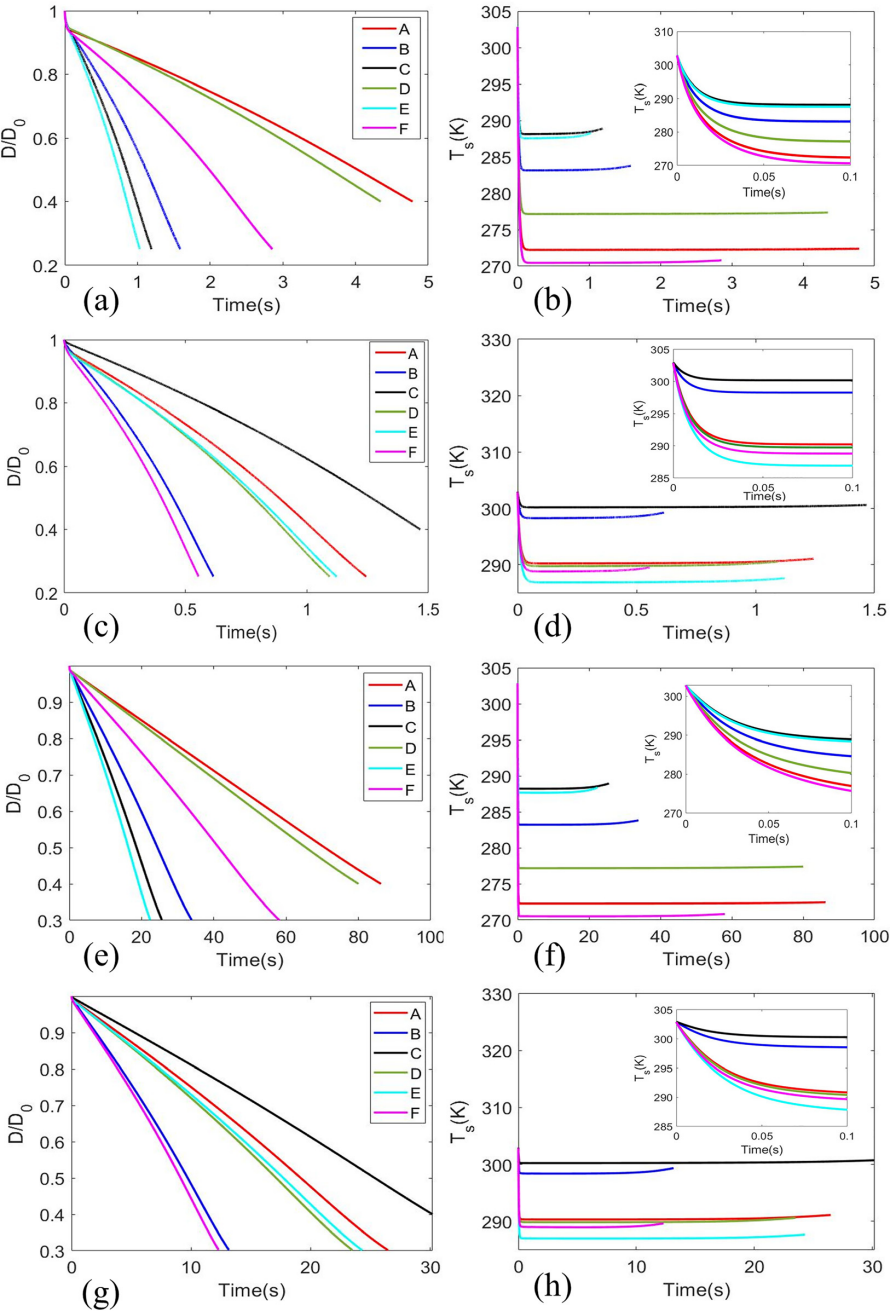
Normalized diameter and droplet surface temperature ( $D_{0}=20\;\mu m$) of cities given in Tables I and II for winter (a) and (b) and summer/monsoon (c) and (d), and ( $D_{0}=100\;\mu m$) of cities for winter (e) and (f) and summer/monsoon (g) and (h).

**FIG. 5.**
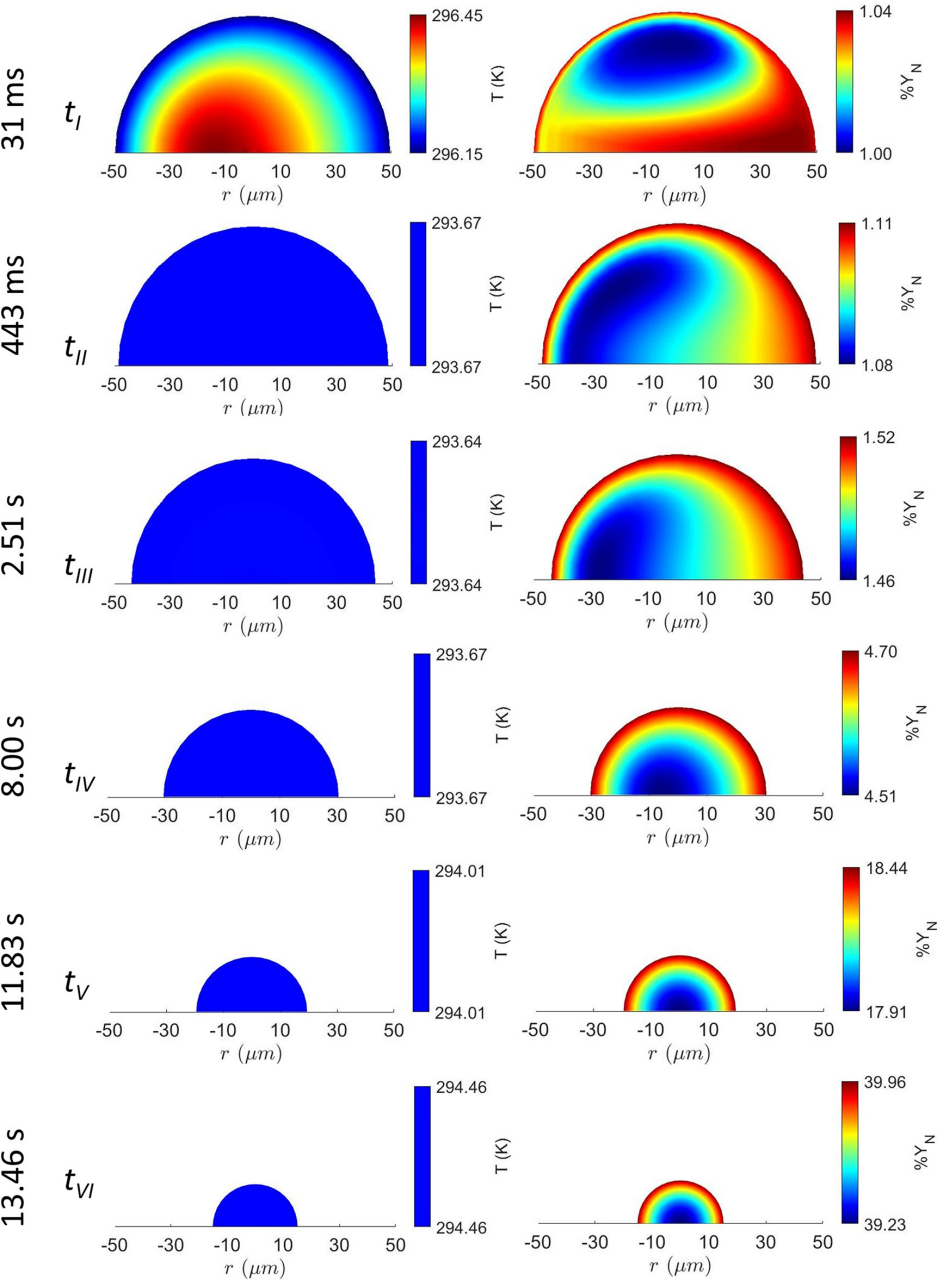
Temperature and salt mass fraction field inside the droplet at different time steps with $D_{0}=100\;\mu m$ at initial conditions of 30 °C droplet surface temperature and 48% relative humidity. Change in the second place of decimal in % $Y_{N}$ represents minimum variation of 1% of initial concentration, which is considered as 1.00%. The stages of t_I_ to t_VI_ are correlated with the stages of Fig. 3(e).

The evolution of normalized diameter $D/D_{0}$ and surface temperature with time t for different initial diameter $D_{0}$ and seasons are represented in Fig. 4 for the cities given in Tables I and II. It is observed that in cities having higher relative humidity, the droplet takes larger time to evaporate. Due to the larger relative humidity, the vapor pressure reduces and reaches a point of equilibrium between the evaporation of water mass from the droplet surface and condensation of water mass from the vapor layer back to the droplet. This delays the evaporation process for which high humidity cities could not evaporate beyond $\frac{D}{D_{0}}∼0.4$. The right-side panel of Fig. 4 shows the evolution of surface temperature of the droplet exhaled initially at 30 °C. The surface temperature rapidly drops to the analogous wet-bulb temperature allowing the heat from the ambient to enter the droplet leading to evaporation. The left and right columns in Fig. 5 show the evolution of temperature and concentration contours, respectively, for an evaporating surrogate respiratory droplet at an ambient condition of T_∞_ = 28 °C and RH_∞_ = 0.48. The initial droplet diameter and temperature were $D_{0}$ = 100 μm and T_s,0_ = 30 °C. The initial solute concentration $Y_{N,0}=0.01$. The primary goal of the 2D model is to understand the temperature and solute concentration distribution within the droplet and ascertain the degree of inhomogeneity of the scalars inside the liquid phase. As the solvent vaporizes from the droplet surface, the solute mass fraction near the surface of the droplet is maximum. Messing *et al.*^47^ proposed that precipitation could occur in various conditions depending on the saturation concentration and temperature. However, in this study, we have considered a homogeneous nucleation phenomenon that assumes a uniform and instantaneous precipitation of salt in the zones that achieved critical supersaturation concentration $Y_{N,S}=0.393.$^44^ Figure 3(e) illustrates the droplet temperature plot with time that depicts the initial transient phase up to 100 ms where rapid cooling of a droplet takes place due to evaporation, while heat is supplied from the center of the droplet ( $t_{I}-t_{II}$). In this region, the temperature contours follow the flow streamlines line to some extent, thereby exhibiting some convection effects due to relative velocity between droplet and jet/puff. However, as the temperature reaches the wet bulb ( $t_{\mathrm{III}}-t_{VI}$), both temperature and concentration contours become concentric exhibiting minimal convection. This is because, at later stages, as the droplet velocity reaches the velocity of the puff cloud, diffusive transport is most dominant. The homogeneous temperature distribution inside the liquid phase is shown in Fig. 5. The temperature distribution contours are plotted for each time step ( $t_{I}-t_{VI}$) denoted in Fig. 3(e) to illustrate this effect. However, the concentration distributions exhibit concentric lines at slightly later stages due to low mass diffusivity compared to thermal diffusivity. However, in the case of spray combustions in liquid-fueled engines and in plasma jets, the convective effects become significant. Such phenomena cause distortion of the spherical symmetry of the temperature distribution inside the droplet.^48–55^ It is also observed that the salt concentration enhances near the droplet surface due to evaporation. As the droplet moves in the air, its size reduces and the surface NaCl concentration reaches the critical supersaturation limit ${\%Y}_{N,S}=39.3\%$. Here, % concentration of salt is presented taking the initial concentration as 1%. Salt accumulation slows down the rate of vaporization significantly and crystallization starts from the droplet surface. We observe similar trends for different ambient conditions.

**FIG. 6.**
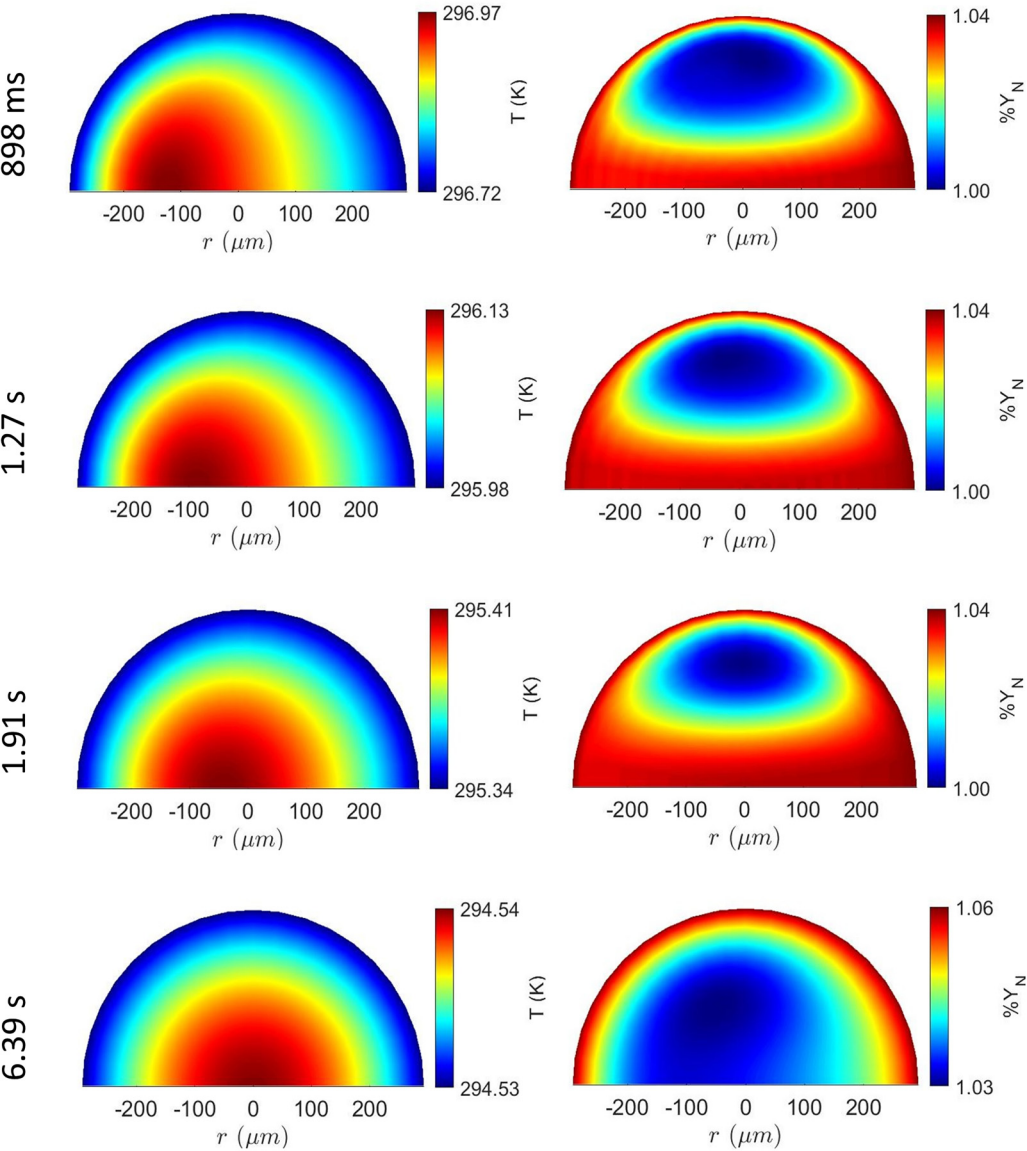
Temperature and salt mass fraction field inside the droplet at different time steps with $D_{0}=593\;\mu m$ at initial conditions of 30 °C droplet surface temperature and 48% relative humidity. Change in the 2nd place of decimal in % $Y_{N}$ represents minimum variation of 1% of initial concentration, which is considered as 1.00%.

However, the model can capture the inhomogeneity in temperature and concentration gradient in bigger droplets as shown in Fig. 6. For droplet of initial diameter $D_{0}$ = 593 μm, the left and right panel shows the temperature and concentration contours, respectively, at ambient temperature of 28 °C and relative humidity 48%. The initial droplet temperature were T_s,0_ = 30 °C with initial solute concentration of ${\%Y}_{N,0}=1\%$. Unlike smaller droplets as shown in Fig. 5, temperature gradient in larger droplets takes longer time of about 6.39 s to attain homogeneity. During this period, the droplet surface temperature reduces due to evaporation and finally attains a wet bulb temperature. This causes rapid cooling of the droplet, while heat is supplied from the center of the droplet. Moreover, both the temperature and concentration contours exhibit stronger convection effects due to relative velocity between the droplet and the ambient (1 m/s). This elucidates the significance of the 2D model to capture such convection effects in larger droplets, which could not be possible in a homogeneous model.

**FIG. 7.**
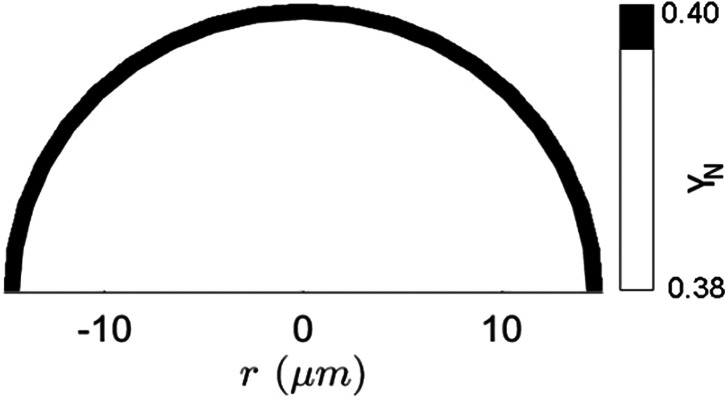
Black Droplet regions with solute concentration $Y_{N,S}>0.393$ with $D_{0}=100\;\mu m$.

Figure 7 reveals the region in which solute concentration $Y_{N,S}>0.393$ in a droplet of initial diameter $D_{0}$ = 100 μm. In this region, the solute concentration becomes higher than the supersaturation concentration, which triggers the precipitation formation. The salt precipitate is uniform along the surface of the droplet as homogeneous nucleation is considered. In a nutshell, low relative velocity and hence low convective effects ensure homogeneous temperature and concentration distribution within the droplet in the 2D model. This model is terminated as the solute concentration reaches the supersaturation concentration. However, to upgrade the pseudokinetic model of crystallization,^4^ the current 2D model can be expanded to understand the nucleation phase inside a droplet.

**FIG. 8.**
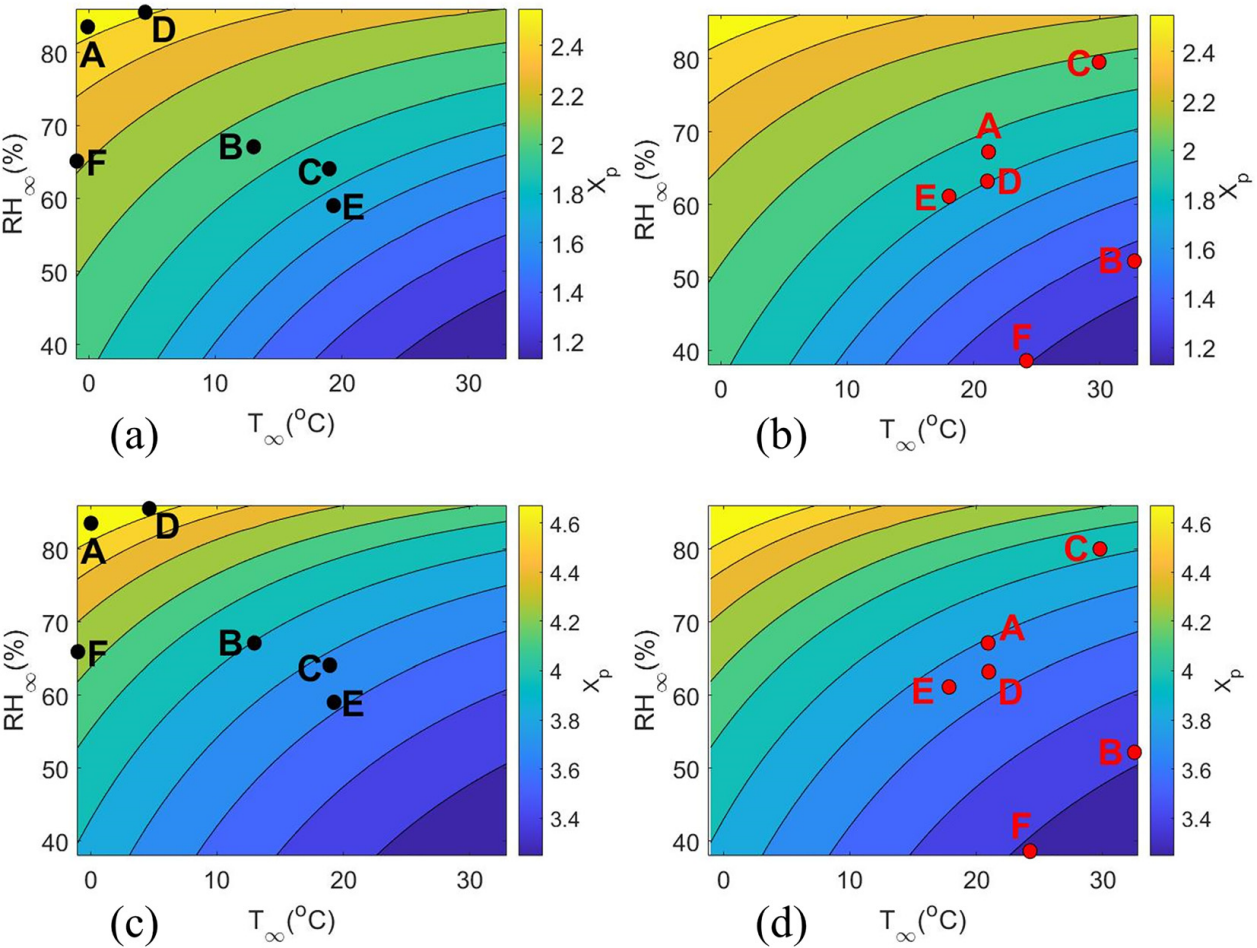
Contours of calculated axial distance $X_{p}$ traveled by the droplet before crystallization in (a) winter (b) summer/monsoon as a function of $T_{\infty }$ and ${RH}_{\infty }$ for $D_{0}=20\;\mu m$, (c) winter and (d) summer/monsoon for $D_{0}=100\;\mu m$. The black and red dots represent different cities from Tables I and II in winter and summer/monsoon, respectively.

Figure 8 gives the distribution of axial distance $x_{p}$ traveled by the droplet in the air, over a wide range of ambient temperature and relative humidity for two different initial droplet diameter ( $D_{0}$ = 20 and 100 μm). For both diameters, interestingly we observe that at high ambient temperature and low relative humidity, the distance traveled by the droplet is minimum. This interprets that the time of evaporation τ must be smaller in cases such as this.^4^ However, for low ambient temperature and high relative humidity, the axial distance $x_{p}$ increases as the time τ, the droplet takes to evaporate is much larger. The contour plots [Figs. 4(a) and 4(c)] are produced for winter season and [Figs. 4(b) and 4(d)] for summer/monsoon. Different cities across the globe are denoted on the contour plots (Tables I and II) for both $D_{0}$. The axial spread of the droplets just prior to nuclei formation in outdoor conditions is observed to be maximum in Berlin and London during the winters, followed by New York, Delhi, and finally Nairobi and Kolkata. However, during summer/monsoon, Kolkata will have the highest axial spread, followed by London, Berlin, Nairobi, and finally New York and Delhi. Once droplets form nuclei, they can remain suspended in the air for hours and can travel much further distance. In cases of such aerosols, the distance traversed by the droplets prior to crystallization/nuclei formation becomes insignificant and largely redundant. However, for diseases where droplets are the principle mode of transmission rather than aerosols, the contour plots and associated distances as shown in Fig. 8 are significant. In addition, for short-range spread of diseases through droplets, the above data may be significantly useful.

## IV. CONCLUSION

In this paper, we have presented a two-dimensional modeling approach that aids to a detailed scientific-based explanation on the transport and translation process in respiratory bio-aerosol (smaller droplets). Respiratory pathogens such as coronavirus, influenza virus, *Mycobacterium tuberculosis*, and *Pseudomonas aeruginosa* exhaled during any respiratory event from an infectious person are carried within these droplets and get transmitted. Therefore, it becomes necessary to understand the evolution of the exhaled droplets characterized by complex interaction of spatiotemporal aerodynamics and evaporation kinetics. Once a droplet cloud is exhaled in any respiratory event, the droplets experience aerodynamical drag, increasing fluid shear rate and causing internal circulations inside the spherical droplet. The main motivation of this approach is to understand detailed flow dynamics in the liquid phase of the droplet while moving in the ambient air. In cases such as turbulent windy conditions causing high relative velocity and greater temperature differences, this model is significantly useful. The transient heat and mass transport equations in liquid phase provide significant knowledge on the internal flow dynamics and concentration gradient. The presence of nonvolatile solutes in the respiratory fluid generates this concentration gradient that enhances at the droplet surface due to evaporation process. The model is simulated until the surface concentration reaches a critical value after which droplet nucleation starts. Moreover, it is observed that the axial spread for bio-aerosols with initial diameter less than 50 *μ*m is higher as they start nucleating in the air and does not settle on the ground. These aerosol particles can float in the air for longer time (hours) enhancing the spread of pathogens. The current model is capable of successfully predicting the evaporation time and axial spread of the droplets in any geographical location on the globe until the point of nuclei formation.

## SUPPLEMENTARY MATERIAL

See the supplementary material showing grid independence study and numerical schemes adopted to solve the equations.

## Data Availability

The data that support the findings of this study are available within the article.
